# Differential expression of miRNAs in enterovirus 71-infected cells

**DOI:** 10.1186/s12985-015-0288-2

**Published:** 2015-04-10

**Authors:** Meng Xun, Chao-Feng Ma, Quan-Li Du, Yan-Hong Ji, Ji-Ru Xu

**Affiliations:** Department of Immunology and Microbiology, Medical School of Xi’an Jiaotong University, Xi’an, 710061 Shaanxi China; Department of Viral Diseases Laboratory, Xi’an Center for Disease Control and Prevention, Xi’an, 710054 Shaanxi China

**Keywords:** Enterovirus 71, Hand, foot and mouth disease, MicroRNA, Microarray

## Abstract

**Background:**

Enterovirus 71 (EV71) is one of the major etiological pathogens of hand, foot and mouth disease (HFMD) and can cause severe cerebral and pulmonary complications and even fatality. MicroRNAs (miRNAs), a class of small non-coding RNA molecules, play an important role in post-transcriptional regulation of gene expression and thereby influencing various physiological and pathological processes. Increasing evidence suggests that miRNAs act as key effector molecules in the complicated pathogen-host interactions. However, the roles of miRNAs in EV71 infection and pathogenesis are not well understood.

**Methods:**

To identify special miRNAs involved in EV71 infection, a microarray assay was performed to study the expression pattern of miRNAs in EV71-infected human rhabdomyosarcoma cells (RD cells) and uninfected RD cells. We further predicted the putative target genes for the dysregulated miRNAs using the online bioinformatic algorithms (TargetScan, miRanda and PicTar) and carried out functional annotation including GO enrichment and KEGG pathway analysis for miRNA predicted targets. Then, the results of microarray were further confirmed by quantitative RT-PCR.

**Results:**

Totally, 45 differentially expressed miRNAs ware identified by microarray, among which 36 miRNAs were up-regulated and 9 were down-regulated. 7166 predicted target genes for the dysregulated miRNAs were revealed by using TargetScan in conjunction with miRanda and PicTar. The GO annotation suggested that predicted targets of miRNAs were enriched into the category of signal transduction, regulation of transcription, metabolic process, protein phosphorylation, apoptotic process and immune response. KEGG pathway analysis suggested that these predicted target genes were involved in many important pathways, mainly including endocytosis and focal adhesion, MAPK signaling pathway, hypertrophic cardiomyopathy, melanogenesis and ErbB signaling pathway. The expression levels of 8 most differentially up-regulated miRNAs and 3 most differentially down-regulated miRNAs were confirmed by qRT-PCR. The expressions of hsa-miR-4530, hsa-miR-4492, hsa-miR-6125, hsa-miR-494-3p, hsa-miR-638, hsa-miR-6743-5p, hsa-miR-4459 and hsa-miR-4443 detected by qRT-PCR were consistent with the microarray data.

**Conclusion:**

These results might extend our understanding to the regulatory mechanism of miRNAs underlying the pathogenesis of EV71 infection, thus strengthening the preventative and therapeutic strategies of HFMD caused by EV71.

## Background

Enterovirus 71 (EV71) is a single-positive-stranded RNA virus belonging to the *Enterovirus* genus of the *Picornaviridae* family, and has become one of the most important neurotropic enteroviruses after the near eradication of poliovirus [1,2]. It is the major causative pathogen of hand, foot and mouth disease (HFMD) which is a common exanthematous and febrile disease in children. But for some patients especially in children under 5 years old, EV71 can cause severe complications, including myocarditis, aseptic meningitis, encephalitis, acute flaccid paralysis, pulmonary oedema or haemorrhage, and even fatality. The underlying mechanisms of serious cerebral and pulmonary complications and even death are not very clear. And up to now, no specific vaccines, effective antiviral agents or therapies against EV71 infection are available [3]. Therefore, the needs of better understanding the mechanism of EV71 pathogenesis are warranted.

Increasing evidence suggests that microRNAs (miRNAs), an abundant class of endogenous small noncoding RNA molecules (~22 nucleotides), modulate gene expression at the posttranscriptional level [4]. miRNAs are processed from the primary transcripts (pri-miRNA) which are subsequently cleaved by Drosha into pre-miRNA of approximately 70 nucleotides and exported to the cytoplasm [5]. In the cytoplasm, cleavage of pre-miRNA by Dicer proteins yields a mature miRNA of approximately 22 nucleotides [6]. Then, the mature miRNAs bind to 3′ untranslated regions (3′ UTR) of target mRNAs, which is able to recruit target mRNA and initiate mRNA degradation or translation inhibition [7]. Therefore, a large number of diverse biological processes, such as cell proliferation, cell cycle, apoptosis, immune response, differentiation, metabolic processes and cellular response to stress are regulated [4,8-10].

It has been demonstrated that miRNAs act as key effector molecules in the complicated interaction network between virus and host [11,12]. On one hand, miRNAs encoded by human cells could directly affect the virus replication. On the other hand, virus could change cell normal functions by influencing cellular gene expressions through miRNAs. To identify cellular miRNAs involved in the host response to EV71 infection and to elucidate the pathogenesis mechanism of EV71 virus, miRNAs profiles in the EV71-infected cells were examined in this study. The differences in expression levels were confirmed by quantitative real-time RT-PCR (qRT-PCR) assay. The computational methods were further performed to identify potential targets of dysregulated miRNAs, With gene ontology (GO) and KEGG biological pathway analysis, several pathways were enriched. These results may enhance our understanding on the prevention and treatment of hand-foot-and-mouth disease caused by EV71.

## Results

### Virus infection and observation of cytopathic effect

**Figure 1:**
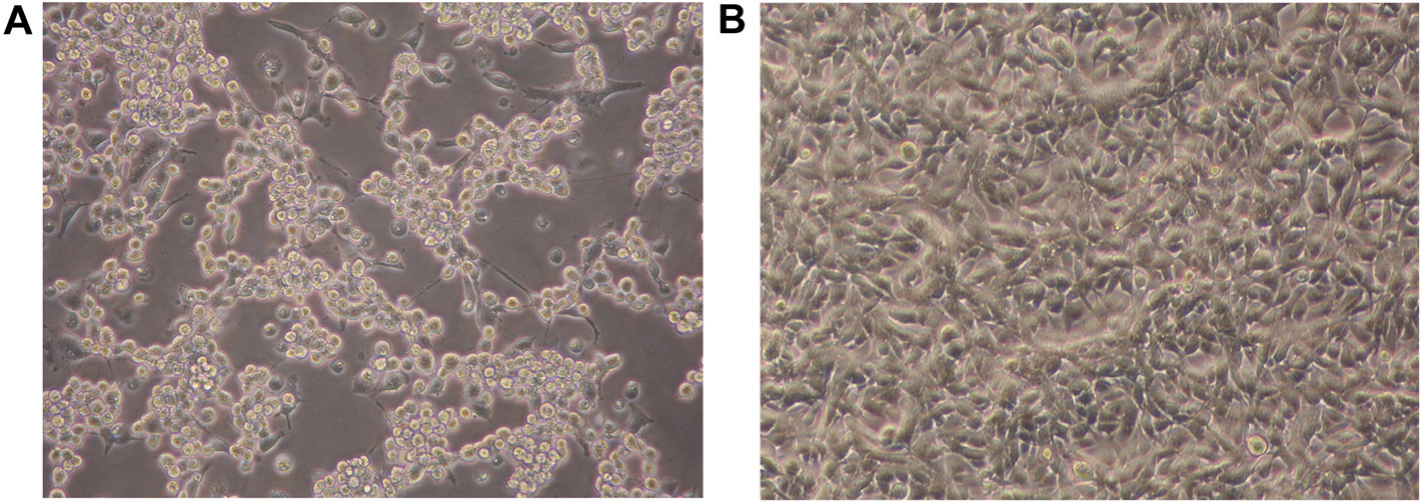
**The morphological changes of EV71-induced cytopathic effect in RD cells were observed under a light microscope at 20× magnification at 48 h p.i..** RD cells were infected with EV71 at a m.o.i. of 0.1. **(A)** EV71-infected RD cells exhibited severe CPE appearance in response to virus replication at 48 h p.i..; **(B)** Uninfected RD cells showed normal morphology.

RD cells which are frequently used to isolate EV71 from clinical specimens are highly susceptible to EV71. After 48 h of infection, EV71 induced a severe CPE in RD cells (Figure 1).

### MiRNA microarray analysis

**Figure 2:**
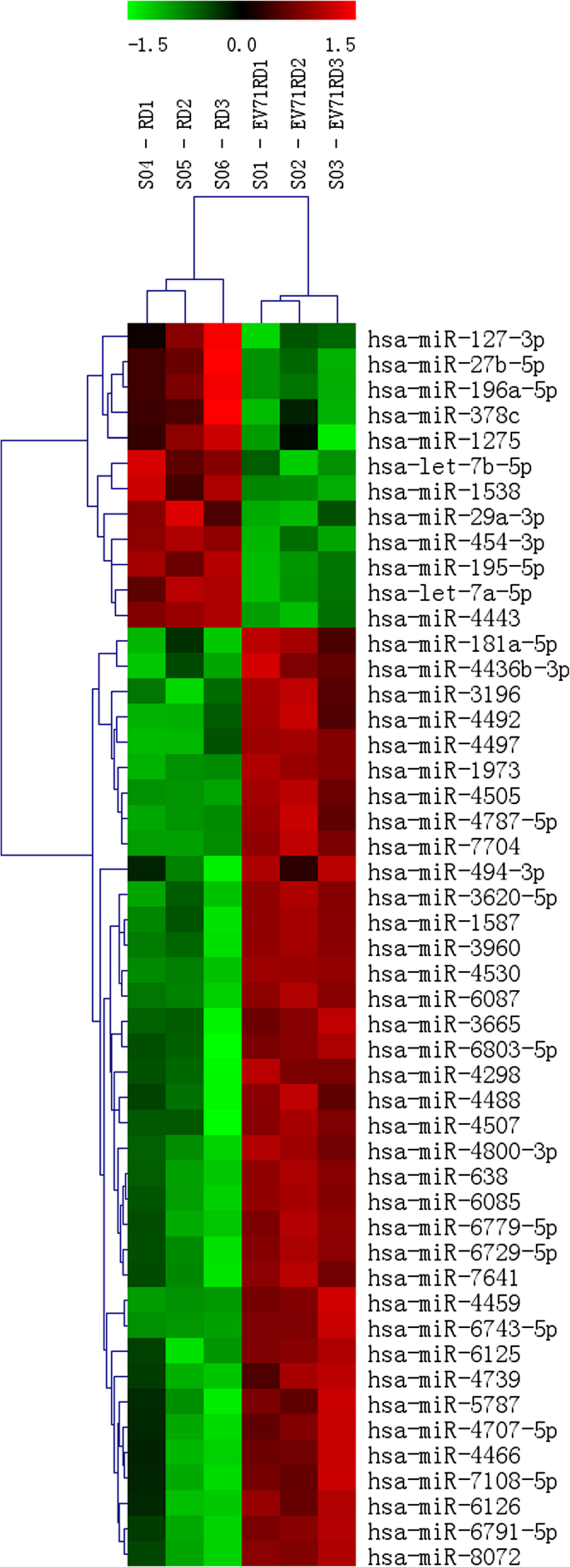
**Heat map and unsupervised hierarchical cluster analysis of the differentially expressed miRNAs in EV71-infected cells and control cells.** Total RNA from EV71-infected RD cells and uninfected RD cells in triplicate was used for microarray. Differentially expressed miRNAs were chosen with a log2 Ratio ≥ 0.5 and ≤ −0.5 and an adjusted *P* value < 0.05. Then columns and rows represent samples and particular miRNAs. The samples were correctly grouped together into EV71-infected cells and control cells according to the expression pattern The miRNA clustering tree is shown on the left. The color scale illustrates the relative expression level of miRNAs. Red color represents that the miRNA has higher expression in EV71-infected cells than in control, while green color represents that the miRNA has lower expression in EV71-infected cells than in control. The codes on the legend are log2-transformed values.

To identify miRNAs involved in EV71 infection and pathogenic mechanism, microarray analysis was performed using total RNA from EV71 infected RD cells and control cells. According to the miRNA profiling, the expression levels of 45 miRNAs were significantly altered in EV71 infected RD cells compared to uninfected RD cells. Among these, 36 miRNAs were up-regulated and 9 were down-regulated. Group-specific signal intensities were shown in Figure 2, and the information of the 45 miRNAs was listed separately in Table 1 with fold changes and *P* values.

**Table 1:** **Summary of the most significantly differentally expressd miRNA**

**miR-name**	**Fold change**	***P*** **values**	**Target sequence (5′ to 3′)**
**Up-regulated miRNAs**			
hsa-miR-4800-3p	28.83	4.19E-03	CAUCCGUCCGUCUGUCCAC
hsa-miR-4530	12.00	4.64E-03	CCCAGCAGGACGGGAGCG
hsa-miR-4492	9.61	7.36E-03	GGGGCUGGGCGCGCGCC
hsa-miR-4507	8.88	3.76E-02	CUGGGUUGGGCUGGGCUGGG
hsa-miR-3620-5p	8.86	1.18E-02	GUGGGCUGGGCUGGGCUGGGCC
hsa-miR-4505	8.72	6.33E-03	AGGCUGGGCUGGGACGGA
hsa-miR-1587	8.54	2.03E-02	UUGGGCUGGGCUGGGUUGGG
hsa-miR-7108-5p	8.19	2.11E-02	GUGUGGCCGGCAGGCGGGUGG
hsa-miR-6791-5p	6.14	2.63E-02	CCCCUGGGGCUGGGCAGGCGGA
hsa-miR-6125	5.71	2.61E-02	GCGGAAGGCGGAGCGGCGGA
hsa-miR-494-3p	5.49	3.58E-02	UGAAACAUACACGGGAAACCUC
hsa-miR-1973	5.11	4.13E-04	ACCGUGCAAAGGUAGCAUA
hsa-miR-6729-5p	4.96	2.12E-02	UGGGCGAGGGCGGCUGAGCGGC
hsa-miR-638	4.61	1.17E-02	AGGGAUCGCGGGCGGGUGGCGGCCU
hsa-miR-3960	4.15	1.55E-02	GGCGGCGGCGGAGGCGGGGG
hsa-miR-6126	4.05	3.70E-02	GUGAAGGCCCGGCGGAGA
hsa-miR-4787-5p	4.04	1.08E-02	GCGGGGGUGGCGGCGGCAUCCC
hsa-miR-8072	4.04	2.07E-02	GGCGGCGGGGAGGUAGGCAG
hsa-miR-6085	3.90	1.49E-02	AAGGGGCUGGGGGAGCACA
hsa-miR-6779-5p	3.85	1.75E-02	CUGGGAGGGGCUGGGUUUGGC
hsa-miR-6087	3.49	1.02E-02	UGAGGCGGGGGGGCGAGC
hsa-miR-4707-5p	3.33	1.81E-02	GCCCCGGCGCGGGCGGGUUCUGG
hsa-miR-7704	3.26	4.44E-03	CGGGGUCGGCGGCGACGUG
hsa-miR-3196	3.25	8.02E-03	CGGGGCGGCAGGGGCCUC
hsa-miR-6743-5p	3.03	6.81E-03	AAGGGGCAGGGACGGGUGGCCC
hsa-miR-4466	2.94	1.88E-02	GGGUGCGGGCCGGCGGGG
hsa-miR-3665	2.77	3.44E-02	AGCAGGUGCGGGGCGGCG
hsa-miR-4497	2.71	1.41E-02	CUCCGGGACGGCUGGGC
hsa-miR-6803-5p	2.62	3.88E-02	CUGGGGGUGGGGGGCUGGGCGU
hsa-miR-5787	2.24	2.21E-02	GGGCUGGGGCGCGGGGAGGU
hsa-miR-7641	2.09	2.70E-02	UUGAUCUCGGAAGCUAAGC
hsa-miR-4298	2.00	3.56E-02	CUGGGACAGGAGGAGGAGGCAG
hsa-miR-4436b-3p	1.99	1.04E-02	CAGGGCAGGAAGAAGUGGACAA
hsa-miR-4459	1.74	8.61E-03	CCAGGAGGCGGAGGAGGUGGAG
hsa-miR-4488	1.63	4.31E-02	AGGGGGCGGGCUCCGGCG
hsa-miR-4739	1.45	1.30E-02	AAGGGAGGAGGAGCGGAGGGGCCCU
**Down-regulated miRNAs**			
hsa-let-7a-5p	0.70	3.40E-03	UGAGGUAGUAGGUUGUAUAGUU
hsa-miR-29a-3p	0.69	1.20E-02	UAGCACCAUCUGAAAUCGGUUA
hsa-miR-127-3p	0.67	4.16E-02	UCGGAUCCGUCUGAGCUUGGCU
hsa-miR-196a-5p	0.59	3.35E-02	UAGGUAGUUUCAUGUUGUUGGG
hsa-miR-378c	0.58	4.53E-02	ACUGGACUUGGAGUCAGAAGAGUGG
hsa-let-7b-5p	0.55	8.67E-03	UGAGGUAGUAGGUUGUGUGGUU
hsa-miR-4443	0.32	1.17E-03	UUGGAGGCGUGGGUUUU
hsa-miR-27b-5p	0.21	4.34E-02	AGAGCUUAGCUGAUUGGUGAAC
hsa-miR-1538	0.16	2.10E-02	CGGCCCGGGCUGCUGCUGUUCCU

### Target genes prediction of the differentially expressed miRNAs and functional analysis

The target prediction was performed for the 45 differentially expressed miRNAs to determine the influence of EV71 infection on potential target mRNAs by combining the results from three online free available algorithms. 6950 predicted target genes of 36 up-regulated microRNAs and 216 predicted target genes of 9 down-regulated microRNAs were found respectively.

**Figure 3:**
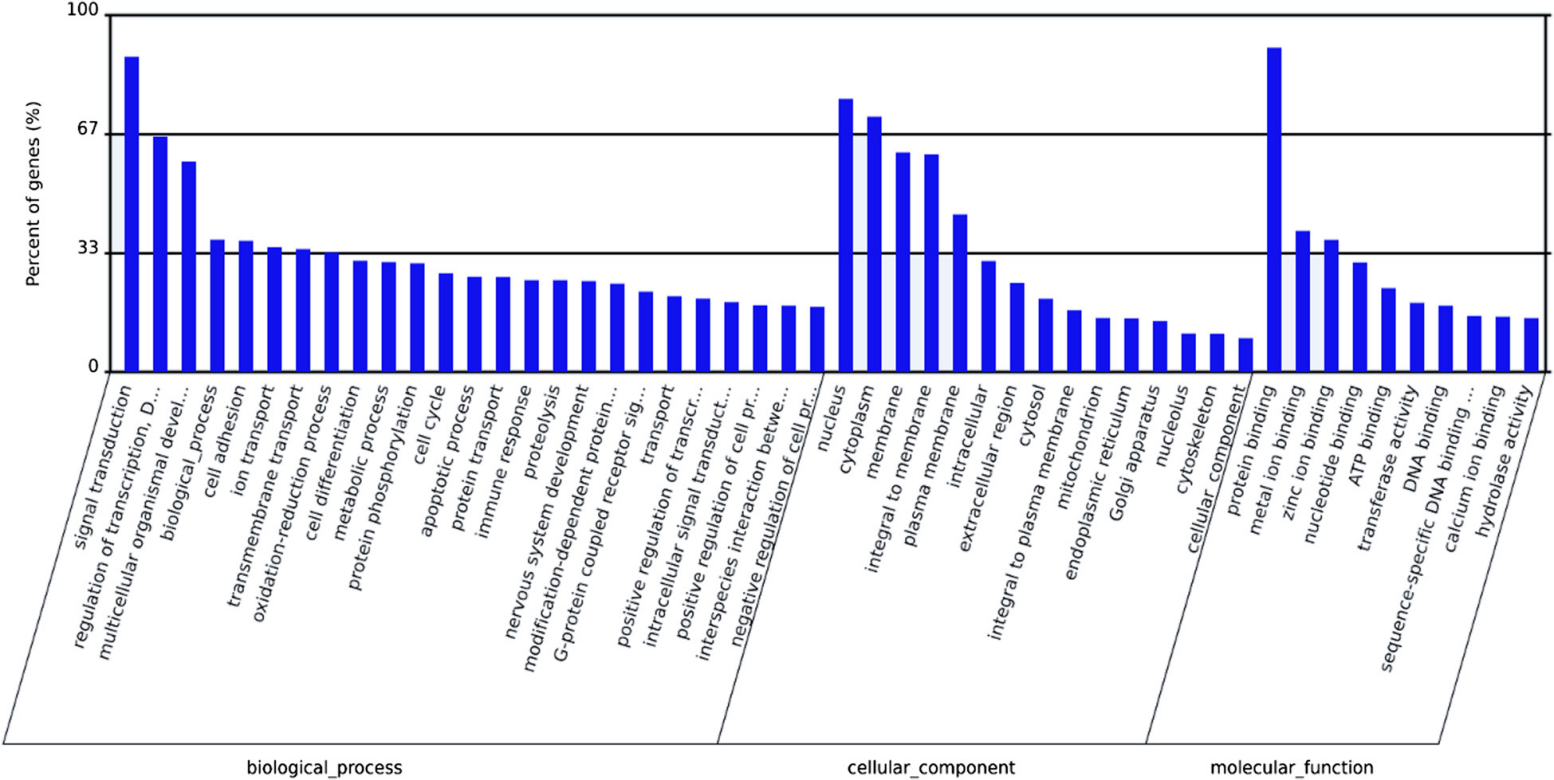
**GO categories and distribution for the predicted miRNA targets related with EV71 infeciton.** The GO terms were sorted by the number of genes in an ascending order from top to bottom. Biological process is enriched in signaling transduction, regulation of transcription, multicellular organismal development, cell adhesion and ion transport. Cellular components are enriched in nucleus, cytoplasm and membrane. Molecular function is enriched in protein binding, metal ion binding and zinc ion binding.

To further evaluate the biological implications of differential miRNA, we appraised miRNA predicted target genes in gene-term enrichment analysis using Gene Ontology (GO) categories. And KEGG (Kyoto Encyclopedia of Genes and Genomes) pathway analysis also was performed by using the DAVID Functional Annotation Chart tool for the targets identified for differentially expressed miRNAs. GO category is composed of biological process, cellular component, and molecular function. Gene-term enrichment analysis revealed that most predicted target genes were involved in GO category “biological process” dealing with signal transduction, regulation of transcription, cell differentiation, metabolic process, protein phosphorylation, cell cycle, apoptotic process and immune response and so on (Figure 3). Within the GO category “cellular component”, the greatest number of predicted target genes had functions associated with nucleus, cytoplasm and membrane (Figure 3). Regarding the GO category “molecular function”, most of the predicted target genes miRNAs were involved in protein binding, metal ion binding and zinc ion binding (Figure 3). These results were indicated that the targets miRNAs were involved in a wide variety of physiological processes.

According to the analysis of enriched KEGG pathways, predicted target genes of differentially expressed miRNAs were related to endocytosis and focal adhesion, MAPK signaling pathway, hypertrophic cardiomyopathy, melanogenesis and ErbB signaling pathway (Table 2). KEGG Pathway analysis illustrated some of the underlying biological processes that may be involved in EV71 infection and might provide useful clues to further research the miRNA targets.

**Table 2:** **KEGG pathway analysis for the predicted miRNA targets related with EV71 infeciton**

**Pathway Id**	**Pathway description**	**S gene number**	**TS gene number**	**B gene number**	**TB gene number**	***P*** **value of Fisher’s exact test**
4144	Endocytosis	158	3220	181	4524	0.000000
4510	Focal adhesion	163	3220	193	4524	0.000008
4010	MAPK signaling pathway	209	3220	257	4524	0.000084
5410	Hypertrophic cardiomyopathy (HCM)	74	3220	84	4524	0.000168
4916	Melanogenesis	86	3220	100	4524	0.000344
4012	ErbB signaling pathway	73	3220	84	4524	0.000490
5214	Glioma	57	3220	64	4524	0.000509
5210	Colorectal cancer	72	3220	83	4524	0.000605
4512	ECM-receptor interaction	70	3220	81	4524	0.000917
4142	Lysosome	95	3220	113	4524	0.000946
5220	Chronic myeloid leukemia	63	3220	73	4524	0.001796
5223	Non-small cell lung cancer	47	3220	53	4524	0.002023
4910	Insulin signaling pathway	109	3220	133	4524	0.002627
5212	Pancreatic cancer	61	3220	71	4524	0.002697
4912	GnRH signaling pathway	78	3220	93	4524	0.003094
4722	Neurotrophin signaling pathway	101	3220	123	4524	0.003313
4130	SNARE interactions in vesicular transport	32	3220	35	4524	0.003616
4520	Adherens junction	62	3220	73	4524	0.004499
4514	Cell adhesion molecules (CAMs)	105	3220	129	4524	0.004825
5414	Dilated cardiomyopathy	72	3220	86	4524	0.004880

### MiRNA expression validation by qRT-PCR

**Figure 4:**
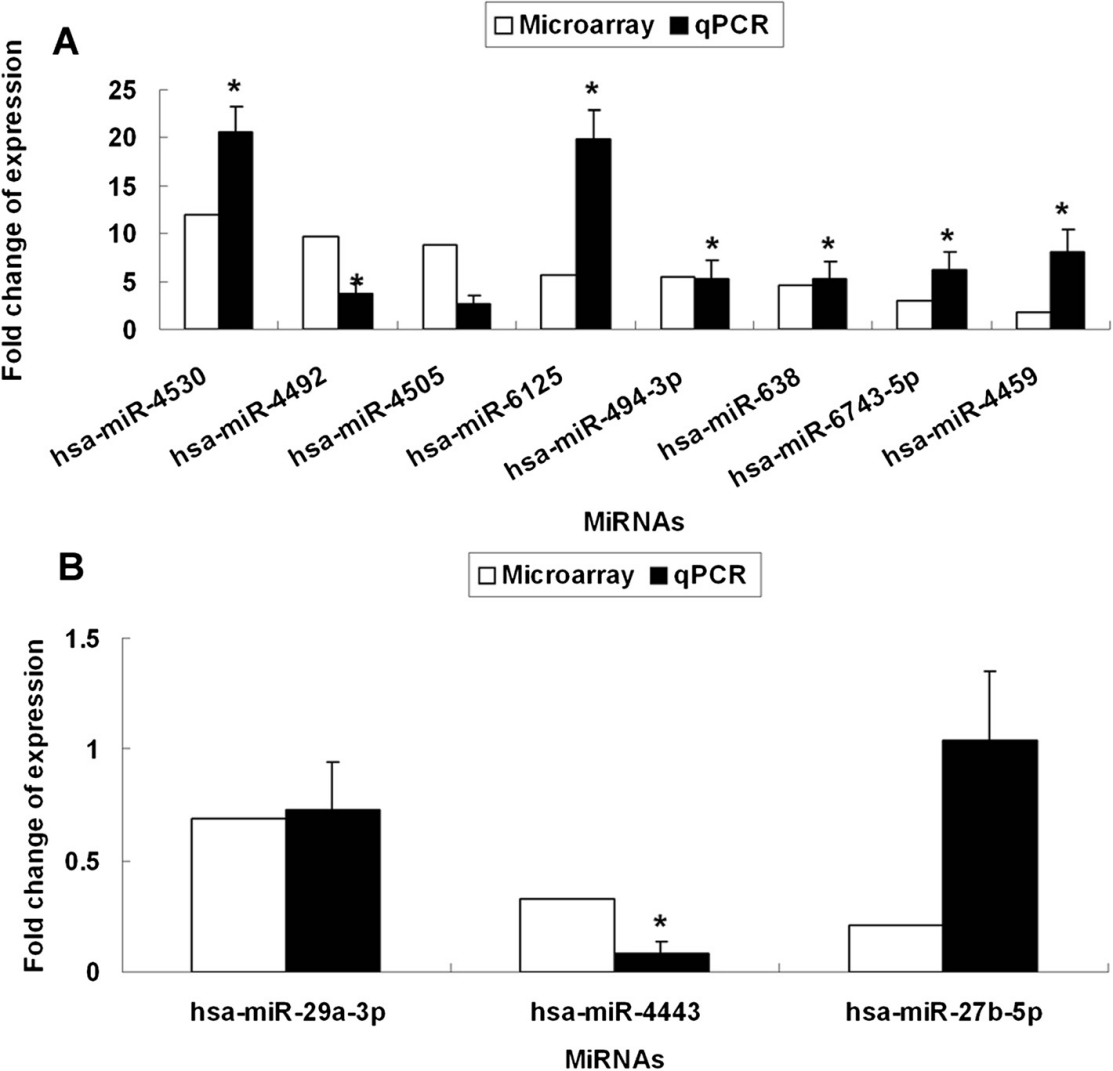
**Validation of the differential expression of 11 miRNAs identified in the microarray by qRT-PCR.** U6 was used as a reference gene. The expression levels of miRNAs in uninfected RD cells were set as 1. The expression levels of most miRNAs detected by qRT-PCR were correlated with those detected by microarray. **(A)** 8 up-regulated miRNAs (hsa-miR-4530, hsa-miR-4492, hsa-miR-4505, hsa-miR-6125, hsa-miR-494-3p, hsa-miR-638, hsa-miR-6743-5p, hsa-miR-4459). **(B)** 3 down-regulated miRNAs (hsa-miR-29a-3p, hsa-miR-4443, hsa-miR-27b-5p). *P < 0.05.

In order to validate the microarray results, the expression levels of 8 most differentially up-regulated miRNAs and 3 most differentially down-regulated miRNAs were examined by qRT-PCR, using the same RNA samples that were used for the microarrays. As shown in Figure 4, the expressions of hsa-miR-4530, hsa-miR-4492, hsa-miR-6125, hsa-miR-494-3p, hsa-miR-638, hsa-miR-6743-5p, hsa-miR-4459 and hsa-miR-4443 detected by qRT-PCR were consistent with the microarray data with significance (*P* < 0.05). The expression levels of hsa-miR-4505 and hsa-miR-29a-3p were not proven to be significant. The expression patterns of hsa-miR-27b-5p was not in line with the microarray results.

## Discussion

Recently, the roles of miRNAs in pathogen-host interactions have been paid more attention than before. Not only the miRNAs encoded by virus but also cellular miRNAs have been proven to participate in the interactions. Through remodelling cellular miRNAs expression, viruses influence cellular microenvironment and metabolism, which are likely to play a very important role in the viral pathogenesis. To date, a few studies have already been done to explore the effects of miRNAs in EV71 infection, inferring that miRNAs may be involved in the pathogenesis of EV71 infection. By using deep sequencing, Cui [13] revealed that certain miRNAs might be essential in the EV71 infection. The research results of Li et al. suggested that miRNA-548 regulates host antiviral response via direct targeting of IFN-λ1 [14]. Using a human colorectal adenocarcinoma cell line (HT29) based EV71 infection model, Lui et al. showed that knockdown of DGCR8, an essential cofactor for microRNAs biogenesis, resulted in a reduction of EV71 replication [15]. Zhang [16] has demonstrated that miR-27a may have antiviral activity against EV71 by inhibiting EGFR. Zheng et al. provned that hsa-miR-296-5p suppressed EV71 replication by targeting the viral genome [17]. And Wen et al. reported that miR-23b inhibited EV71 replication by down-regulating EV71 VPl protein [18]. Recently, Bian et al. [19] compared the miRNA profiles between interferon (IFN)-α or IFN-γ treated RD cells and EV71-infected RD cells. miR-124 and miR-491-3p were regulated in opposite manners by the IFNs and EV71. Wnt signaling cascade, platelet-derived growth factor receptor (PDGFR)/PDGF, phosphatidylinositol 3-kinase(PI3K) and Jun N-terminal kinase (JNK)/mitogen-activated protein kinase (MAPK) were predicted in both EV71 infection and IFN treatment. These results indicated that cellular miRNAs participated in the virus life cycle as critical host factors by targeting the downstream genes or signaling pathways. The identified miRNAs and signal transduction patyway would be helpful to understand the interaction between the virus and host.

In our present study, a comprehensive miRNA profile was performed in EV71-infected RD cells through microarray assay to identify cellular miRNAs and downstream signaling pathway involved in the host response to EV71 infection. 36 up-regulated and 9 down-regulated miRNAs were found. By using qRT-PCR, the expression level of 11 miRNAs were identified. Among them, the expression patterns of 7 up-regulated (hsa-miR-4530, hsa-miR-4492, hsa-miR-6125, hsa-miR-494-3p, hsa-miR-638, hsa-miR-6743-5p, hsa-miR-4459) and 1 down-regulated miRNA (hsa-miR-4443) were consistent with the microarray data.

Among the 8 miRNAs, some miRNAs have been reported in other research field. Hsa-miR-4530 was validated as a miRNA marker that differentiated pancreato-biliary cancers from other clinical conditions including healthy controls, non-malignant abnormalities, and other types of cancers [20]. Hsa-miR-4459 could decrease the expression of its targets, CDC20B and ATG13, and thus altered stemness via cell cycle and autophagy in human embryonic stem cells [21]. Hsa-miR-494-3p, a well-known miRNA, plays different roles in different malignancies and was found to be implicated in multiple cell processes including cell proliferation, apoptosis, and invasion. For example, miR-494-3p was found to be act as a cancer gene that could promote glioma cell proliferation through the down-expression of PTEN, a tumor-suppressor gene [22,23]. While miR-494 acts as an antioncogene in gastric carcinoma by targeting c-myc in gastric cancer [24,25]. Furthermore, hsa-miR-638 has previously been reported to be associated with virus infection. Up-expression of hsa-miR-638 was identified by microarray technology in chikungunya virus infection in human cells [26]. Kumar et al. [27] reported hsa-miR-638 decreased the levels of HBV transcripts or HBV gene products. Liu et al. [28] demonstrated inhibition of hsa-miR- 638 slightly increased HCV entry during in vitro acute HCV infection. But all of the 8 miRNAs including hsa-miR-4530, hsa-miR-4492, hsa-miR-6125, hsa-miR-494-3p, hsa-miR-638, hsa-miR-6743-5p, hsa-miR-4459 and hsa-miR-4443 were found to be related with EV71 infection for the first time. This may provide new clues for EV71 infection research.

According to the result of GO analysis, the predicted target genes were involved in signal transduction, regulation of transcription, cell differentiation, metabolic process, protein phosphorylation, cell cycle, apoptotic process and immune response et al.. These biological processes are reported to be crucial in the interplay between host and virus. As for biological pathways, endocytosis and focal adhesion, MAPK signaling pathway and ErbB signaling pathway et al. were among the enriched pathways of the predicted target genes.

Mitogen-activated protein kinase (MAPK) are a family of protein kinases responsible for phosphorylating serine and threonine in many proteins [29]. It is widely conserved in eukaryotes and involved in many cellular functions such as inflammation, cell proliferation, differentiation, movement and death [30-32]. Three major MAPK signaling pathways have been identified, including extracellular regulated kinases (ERK1/2), JNK(JNK1/2) and p38 MAPK (p38 α/β/γ/δ). It has been reported that JNK1/2 and p38 MAPK signal pathways plays an important role in response to infection and replication of human immunodeficiency virus type 1, encephalomyocarditis virus, coxsackievirus B3, hepatitis C virus, herpes simplex virus 1, and the severe acute respiratory syndrome coronavirus [33-36]. The diverse effects of activates signaling cascades include induction of apoptosis in infected cells and enhancement of viral replication. MEK1 is crucial in the ERK signaling cascade and is required to promote EV71 replication [37]. EV71 infection induces the up-regulated gene expressions of MAPK signaling pathway such as ERK, JNK and PI3K/AKT, which may be associated with the secretions of inflammatory cytokines and host cell apoptosis, which might be implicated in CNS inflammation and disorders such as encephalitis or meningitis [38]. Similarly, Tung et al. [39] domenstrated that cellular ERK levels have been shown to be the major causal factor for upregulation of cyclooxygenase-2 (COX-2) expression induced by EV71 infection which may participate in EV71-induced CNS damage. COX-2 and its metabolite, prostaglandin E2 (PGE2), which are considered a tumor biomarker [40], are up-regulated by EV71 infection via the activated c-Src/PDGFR/PI3K/Akt/p42/p44 MAPK/AP1 and NF-κB pathways in rat brain astrocytes [41]. These evidence together with our study supports the notion that MAPK signalling cascade as key moleculars in EV71 replication cycle and pathogenesis.

As well known, the miRNA targets predicition merely by the means of bioinformatics is not enough. MiRNA targets should be verified by further experiments, such as miRNA transfection or knockdown and luciferase assay. Therefore, our next task is to carry out these experiments to confirm the miRNA targets which were predicted in this present study. If the function of differentially expressed miRNAs and their corresponding predicted target genes have been identified, it will improve the protection and treatment strategies of EV71 infection.

## Materials and methods

### Cell culture, virus infection and observation of cytopathic effect

Human rhabdomyosarcoma cells (RD cells; ATCC CCL-136, Manassas, VA) were grown in Dulbecco’s modified Eagle’s medium (DMEM; HyClone, Logan, UT) supplemented with 10% fetal bovine serum (FBS; Gibco, USA) plus 100 U/ml penicillin and 100 μg/ml streptomycin at 37°C with 5% CO_2_. When the cells had grown to 90% confluence, cells were infected with EV71 (Human enterovirus 71 strain 87–2008 isolated and identified from Shaanxi Province, China, in 2010, Genbank accession no. HM003207) at a m.o.i. of 0.1 and maintained in medium containing 2% fetal calf serum following 1 h adsorption at 37°C. After infection, the morphological changes of virus-induced cytopathic effect were observed and photographed under microscopy.

### Total RNA extraction and quality control

EV71-infected RD Cells and uninfected RD cells in triplicate were harvested at 48 h postinfection and resuspended in 1 ml of TRIzol reagent (life technology), strongly shaken and stored at −20°C. Total RNA, including small RNA, was extracted using Total RNA Kit (LC Sciences, USA) according to the manufacturer’s protocol. After purification, the concentration of RNA samples was determined by measuring the absorbance at 260 and 280 nm with the NanoDrop 1000. And the integrity was assayed by Agilent 2100 Bioanalyzer (Agilent Technologies, USA). Only cases with RNA Integrity Numbers (RIN) ≥7–10 were further used.

### MicroRNA microarray assay

MiRNA microarray, covering all miRNAs in the miRBase database (v.20.0) (www.mirbase.org), was conducted by LC Sciences (Houston, TX, USA) to study the expression profiling. 5 ug of RNA from each sample was size fractionated by using an YM-100 Microcon centrifugal filter (Millipore, Bedford, MA, USA). The small RNAs(<300 nt) were 3′-extended with a poly(A) tail using poly(A) polymerase. An oligonucleotide tag was then ligated to the poly(A) tail for later fluorescent dye staining. Hybridization was performed overnight on a μParaflo microfluidic chip using a micro-circulation pump (Atactic Technologies) [42]. On the microfluidic chip, each detection probe consisted of a chemically modified nucleotide coding segment complementary to target microRNA from miRBase and a spacer segment of polyethylene glycol to extend the coding segment away from the substrate. The detection probes were made by in situ synthesis using PGR (photogenerated reagent) chemistry. The hybridization melting temperatures were balanced by chemical modifications of the detection probes. The hybridization buffer was 100 μL 6 × SSPE buffer (0.90 M NaCl, 60 mM Na_2_HPO_4_, 6 mM EDTA, pH 6.8) containing 25% formamide at 34°C. After RNA hybridization, tag-conjugating Cy3 dye were circulated through the microfluidic chip for dye staining. Fluorescence images were collected using a laser scanner (GenePix 4000B, Molecular Device) and digitize analysis was performed using Array-Pro image analysis software (Media Cybernetics, Bethesda, MD). Data were analyzed by first subtracting the background and then normalizing the signals using a LOWESS filter (Locally-weighted Regression) [43]. The differentially expressed miRNAs were defined using the ratio of detected signals log2-fold changes [log2(infected/control)] and the Student’s t-test was used to calculate *P* values. Those with a log2 ratio >0.5 or ≤ −0.5 and *P* values <0.05 were considered as differentially expressed miRNAs. The cluster analysis based on the relative expression levels of miRNAs was also carried out.

### Target gene prediction, GO enrichment and KEGG pathway analysis

Prediction of putative miRNA targets was performed by using the online softwares, TargetScan (http://www.targetscan.org/) in conjunction with miRanda (http://www.microrna.org/microrna/home.do) and PicTar (http://pictar.mdc-berlin.de/). The enriched GO (http://www.geneontology.org/) terms and KEGG pathways database (http://www.genome.jp/kegg/) for the predicted target genes of the differentially expressed miRNAs were identified by using DAVID (The Database for Annotation, Visualization and Integrated Discovery; http://david.abcc.ncifcrf.gov/) gene annotation tool [44]. Fisher’s two-side exact test and Chi-square test were used to classify the GO categories and KEGG pathway categories, and the false discovery rate (FDR) was also calculated to correct the *P* values. We chose only GOs and the enriched pathways that had a *P* value of <0.05 and a FDR of < 0.05. The result may reveal the functions, metabolic pathways or signal transduction pathways significantly associated to predicted targets.

### Validation of microarray data with qPCR analysis

Validations of differentially expressed miRNAs were carried out by qPCR with SYBR green [45]. 8 of the most significantly up-regulated miRNAs (hsa-miR-4530, hsa-miR-4492, hsa-miR-4505, hsa-miR-6125, hsa-miR-494-3p, hsa-miR-638, hsa-miR-6743-5p and hsa-miR-4459) and 3 of the most significantly down-regulated microRNAs (hsa-miR-29a-3p, hsa-miR-4443, hsa-miR-27b-5p) were selected as representatives for confirmation. Total RNA, which was used for microarray, was polyadenylated and reverse-transcribed with a poly(T) adapter into cDNAs following the manufacturer’s directions with All in One^TM^ miRNA qRT-PCR detection kit (GeneCopoeia). For real-time PCR, the miRNA-specific forward primer and the Universal Adaptor PCR Primer as the reverse primer were used. All of primers were purchased from GeneCopoeia (Rockville, Md, USA) and the miRNA-specific forward primers are listed in Table 3. Real-time PCR was runing on Stratagene mx3005p (Agilent Technologies, USA) in triplicate for each sample. The relative amount of each miRNA was normalized against U6 by the 2^-ΔΔCt^ method.

**Table 3:** **Primers used for qRT-PCR**

**Primer name**	**Forward primer sequence (5′–3′)**	**Product size (bp)**
**Up-regulated miRNAs**		
hsa-miR-4530	CAGGACGGGAGCGAAAA	66
hsa-miR-4492	CTGGGCGCGCGCCAAA	66
hsa-miR-4505	CTGGGCTGGGACGGAAAA	68
hsa-miR-6125	GCGGAGCGGCGGAAAA	67
hsa-miR-494-3p	GAAACATACACGGGAAACCTCAA	74
hsa-miR-638	GGGCGGGTGGCGGCCTAAA	69
hsa-miR-6743-5p	AGGGACGGGTGGCCCA	69
hsa-miR-4459	GCGGAGGAGGTGGAGAAA	68
**Down-regulated miRNAs**		
hsa-miR-29a-3p	CACCATCTGAAATCGGTTAA	72
hsa-miR-4443	TGGAGGCGTGGGTTTTAAA	69
hsa-miR-27b-5p	AGAGCTTAGCTGATTGGTGAACAA	75

### Statistical analysis

All statistical analysis was performed using the SPSS 17.0 statistical software (SPSS Inc., Chicago, IL, USA). The difference between two groups was determined by a two-tailed Student’s *t* test, with *P* values of <0.05 considered to be statistically significant.
